# Reasons Why Patients Are Turned Down From Treatment at a Personality Disorder Service: Implications for Referrers and Personality Disorder Services

**DOI:** 10.1192/bjo.2024.458

**Published:** 2024-08-01

**Authors:** Suvitha Krishnan, Kemal Ibrahim, Josephine Agyeman, Harry Reid, Tennyson Lee

**Affiliations:** 1 East London NHS Foundation Trust, London, United Kingdom; 2 Barnet Enfield and Harringey NHS Foundation Trust, London, United Kingdom

## Abstract

**Aims:**

Patients referred to a Personality Disorder (PD) Service are frequently not offered treatment. This has profound implications for patients (who feel dismissed or rejected), referrers (who are perplexed as they have clearly diagnosed a PD) and the PD services themselves (their raison d'etre being to treat PD patients). A systematic search identified no literature on reasons for non-acceptance. This study aimed to describe reasons for not offering therapy in patients, after a specialist assessment.

**Methods:**

We conducted a case series of 50 patients assessed in a specialist PD service. We collected data from routine service notes, using thematic analysis to identify categories of the reasons identified for treatment unsuitability.

**Results:**

Reasons for assessing treatment unsuitability (in descending order) were:

1. (20%) – Lack of engagement (e.g. repeated non-attendance of appointments) and motivation to change (e.g. externalising all responsibility, or believing they completely lacked agency in their actions).
2. (18%) – Extremely harmful substance misuse or dependence.
3. (13%) – The underlying diagnosis (e.g. not meeting diagnostic criteria for a personality disorder or a severe psychopathy) and level of severity (e.g. too mild for a specialist service).
4. (11%) – Identified areas of psychological work has very little to no relation to interpersonal difficulties or relationships.
5. (11%) – A comorbid eating disorder (e.g. BMI < 17.5).
6. (9%) – Another service identified as being more appropriate (e.g. another psychological service).
7. (8%) – Risk of aggression to the therapist.
8. (5%) – Comorbid axis I disorder being the primary problem.
9. (5%) – Extreme self-harming behaviours requiring crisis interventions.

**Conclusion:**

Referrers

1. To accept that many patients with PD will fail to actively engage in psychotherapy.
2. To consider whether severity is of a level requiring specialist PD treatment; or if the patient needs a forensic psychotherapy service rather than a non-forensic PD service.
3. To consider whether the comorbid conditions (e.g. dependent alcohol use) are in fact the primary diagnosis and thus require treatment before the PD service intervention.

PD services

1. Need to develop novel interventions to help patients become more active and engaged in the assessment and thus progress onto treatment.
2. Need to inform referrers on their criteria for not offering treatment, allowing referrers the ability to gauge more accurately when to refer the patient.

